# Supplementary material for: Resveratrol and Its Analogue 4,4′-Dihydroxy-trans-stilbene Inhibit Lewis Lung Carcinoma Growth In Vivo through Apoptosis, Autophagy and Modulation of the Tumour Microenvironment in a Murine Model
Source: Biomedicines. 2022 Jul 25;10(8):1784. doi: 10.3390/biomedicines10081784 (PMC9332680; doi:10.3390/biomedicines10081784)
Supplement: Supplementary file 1 [file biomedicines-10-01784-s001.zip › biomedicines-1806499-Supplementary.pdf]

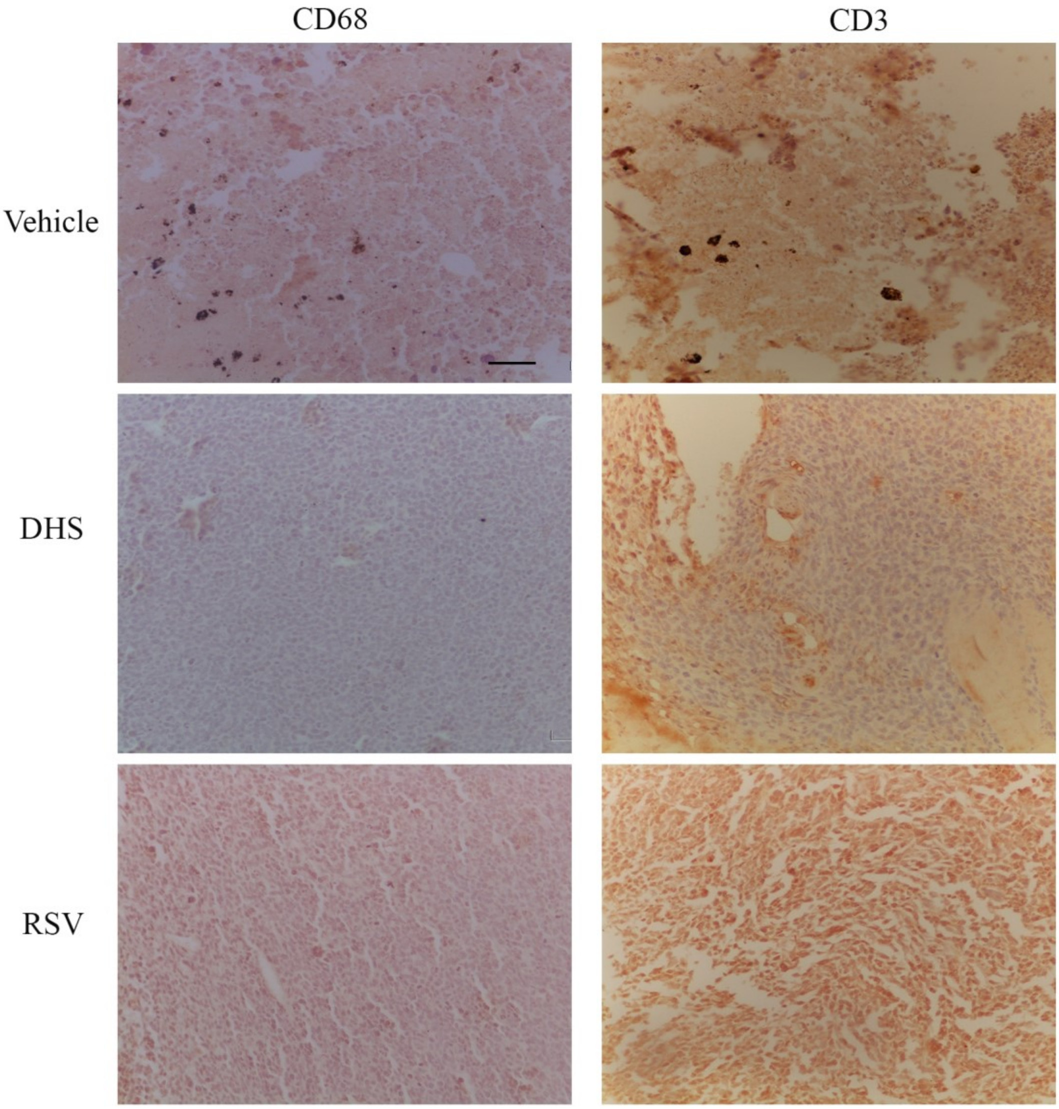

Figure S1. Representative images of CD68 and CD3 immunostaining in vehicle, DHS, and RSV tumour masses (scale bar = 100 μm; 20× magnification).
